# Improvement of sabinene tolerance of *Escherichia coli* using adaptive laboratory evolution and omics technologies

**DOI:** 10.1186/s13068-020-01715-x

**Published:** 2020-04-24

**Authors:** Tong Wu, Jinfeng Liu, Meijie Li, Ge Zhang, Lijuan Liu, Xing Li, Xiao Men, Mo Xian, Haibo Zhang

**Affiliations:** 1 CAS Key Laboratory of Biobased Materials, Qingdao Institute of Bioenergy and Bioprocess Technology, Chinese Academy of Sciences, No. 189 Songling Road, Laoshan District, Qingdao, 266101 China; 2 University of Chinese Academy of Sciences, Beijing, 100049 China; 3 School of Life Science and Engineering, Lanzhou University of Technology, Lanzhou, 730050 China

**Keywords:** *Escherichia coli*, Sabinene tolerance, Adaptive laboratory evolution, Genome resequencing, Transcriptome sequencing, *ybcK*/*ygiZ*/*scpA*

## Abstract

**Background:**

Biosynthesis of sabinene, a bicyclic monoterpene, has been accomplished in engineered microorganisms by introducing heterologous pathways and using renewable sugar as a carbon source. However, the efficiency and titers of this method are limited by the low host tolerance to sabinene (in both eukaryotes and prokaryotes).

**Results:**

In this study, *Escherichia coli* BL21(DE3) was selected as the strain for adaptive laboratory evolution. The strain was evolved by serial passaging in the medium supplemented with gradually increasing concentration of sabinene, and the evolved strain XYF(DE3), which exhibited significant tolerance to sabinene, was obtained. Then, XYF(DE3) was used as the host for sabinene production and an 8.43-fold higher sabinene production was achieved compared with the parental BL21(DE3), reaching 191.76 mg/L. Whole genomes resequencing suggested the XYF(DE3) strain is a hypermutator. A comparative analysis of transcriptomes of XYF(DE3) and BL21(DE3) was carried out to reveal the mechanism underlying the improvement of sabinene tolerance, and 734 up-regulated genes and 857 down-regulated genes were identified. We further tested the roles of the identified genes in sabinene tolerance via reverse engineering. The results demonstrated that overexpressions of *ybcK* gene of the DLP12 family, the inner membrane protein gene *ygiZ*, and the methylmalonyl-CoA mutase gene *scpA* could increase sabinene tolerance of BL21(DE3) by 127.7%, 71.1%, and 75.4%, respectively. Furthermore, scanning electron microscopy was applied to monitor cell morphology. Under sabinene stress, the parental BL21(DE3) showed increased cell length, whereas XYF(DE3) showed normal cell morphology. In addition, overexpression of *ybcK*, *ygiZ* or *scpA* could partially rescue cell morphology under sabinene stress and overexpression of *ygiZ* or *scpA* could increase sabinene production in BL21(DE3).

**Conclusions:**

This study not only obtained a sabinene-tolerant strain for microbial production of sabinene but also revealed potential regulatory mechanisms that are important for sabinene tolerance. In addition, for the first time, *ybcK, ygiZ*, and *scpA* were identified to be important for terpene tolerance in *E. coli* BL21(DE3).

## Background

Terpenoids is the largest class and the most widely distributed secondary metabolites that have been discovered to date [1]. These compounds are widely used in medicine, food additives, perfume, energy industry, and many other fields [2, 3]. Sabinene, a bicyclic monoterpene, is also widely applied in the above fields [4, 5]. Sabinene has the potential to serve as a feedstock for advanced biofuels due to its high energy density, low freezing point, and high flash point [6], and can be used as additive or substitute of adamantane, norbornene, and other chemicals that are used in the manufacture of high-performance and high-density aviation fuel [7, 8]. The chemical catalysis-based synthetic route for sabinene had already been established by organic chemists in last century [9]. However, complexity of the reaction system and high cost of the catalyst prevented the scale-up of the synthetic route at industrial levels [4]. Currently, sabinene is mainly isolated from plants which greatly limits the large-scale production and application of these compounds due to its low content in plant materials [10].

**Fig. 1:**
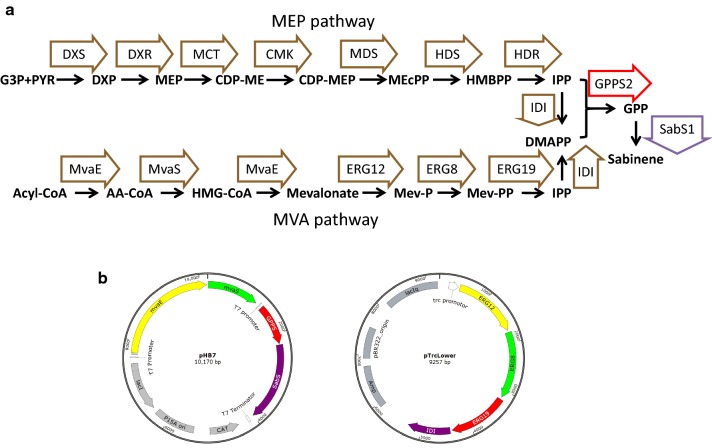
Synthetic pathway of sabinene. **a** Flow chart of sabinene synthetic pathway. MEP pathway intermediates: DXP, 1-deoxy-d-xylulose 5-phosphate; MEP, 2-C-methyl-d-erythritol-4-phosphate; CDP-ME, 4-(cytidine-5′-diphospho)-2-C-methyl-d-erythritol; CDP-MEP, 2-phospho-4-(cytidine-5′-di-phospho)-2-C-methyl-d-erythritol; ME-cPP, 2-C-methyl-d-erythritol 2,4-cyclodiphosphate; HMBPP, 4-hydroxy-3-methylbut-2-enyl diphosphate; IPP, isopentenyl diphosphate; DMAPP, dimethylallyl diphosphate. Enzymes in MEP pathway: DXS, DXP synthase; DXR, DXP reductoisomerase; MCT, CDP-ME synthase; CMK, CDP-ME kinase; MDS, ME-cPP synthase; HDS, HMBPP synthase: HDR, HMBPP reductase; IDI, IPP isomerase. MVA pathway intermediates: Acyl-CoA, acetyl-CoA; AA-CoA, acetoacetyl-CoA; HMG-CoA, hydroxymethylglutaryl-CoA; Mev-P, mevalonate-5-phosphate; Mev-PP, mevalonate pyrophosphate; IPP, isopentenyl pyrophosphate; DMAPP, dimethylallyl pyrophosphate. Enzymes in MVA pathway: MvaE, acetyl-CoA acetyltransferase/HMG-CoA reductase; MvaS, HMG-CoA synthase; ERG12, mevalonate kinase; ERG8, phosphomevalonate kinase; ERG19, mevalonate pyrophosphate decarboxylase; IDI, IPP isomerase. GPPS2, geranyl diphosphate from *Abies grandis*; SabS1, sabinene synthase from *Salvia pomifera*. **b** Schematic diagram of plasmids pHB7 and pTrcLower

Along with the development of synthetic biology and the increasing demand for green and sustainable energy, using renewable sugars as raw materials and genetically modified microorganisms as hosts to produce desired products is attracting more attentions [11, 12]. Biosynthesis of sabinene has been accomplished in engineered *Escherichia coli* and *Saccharomyces cerevisiae* which usually involves the mevalonate (MVA) pathway and/or the methylerythritol 4-phosphate (MEP) pathway [6, 13, 14]. Like for the biosynthesis of many other terpenoids, the two key C5 precursors for sabinene biosynthesis, isopentenyl pyrophosphate (IPP) dimethylallyl and pyrophosphate (DMAPP), can be synthesized from the MVA pathway or the MEP pathway [4]. Subsequently, geranyl diphosphate (GPP) is synthesized through condensation of DMAPP and IPP catalyzed by GPP synthase (GPPS). Then, sabinene is produced from GPP catalyzed by sabinene synthase (SabS) (Fig. 1a).

Most studies on the engineering of microbes to produce various chemicals by metabolic engineering or synthetic biology focused on improving the metabolic efficiency of host strains [15, 16]. However, quite a lot of the fermentation products are organic solvents which exhibit a certain degree of toxicity against the host cells [17–20]. Many of these products exert stress on host strains, which inhibits cell growth and biosynthesis of the product. This problem was apparent in the development of sabinene-producing strains and prevented industrialization of the process [6]. Similar to its analog terpinene, sabinene might insert into cell membrane and widen the gap between the phospholipid molecules, causing loss of stability of membrane proteins and leading to K^+^ leakage, cell lysis, and death [21, 22]. Therefore, improvement of sabinene tolerance of host strains is urgently required.

Adaptive laboratory evolution (ALE) is a frequently used method in metabolic engineering for strain development and optimization, which uses selective pressure as a driving force for the selection of mutants with favorable phenotypes [23, 24]. It has been demonstrated to be a simple and effective strategy in enhancing tolerance and improving productivity of strains to organic solvents, and combination of ALE with next-generation sequencing and modern omics technologies enable researchers to gain insight into the genetic basis and molecular mechanism of dynamic adaptation. For example, ALE was successfully performed in *E. coli* to improve its tolerance to isopropanol (IPA) and mutations contributed to IPA tolerance were identified through genome resequencing [25]. The evolved strain of *S. cerevisiae* by ALE showed significantly enhanced tolerance to limonene and monoterpenes-containing biojet fuels, and genes/proteins involved in phenotypic changes were identified through genome resequencing, transcriptomics, and proteomics analyses [26].

In our previous work, an *E. coli* BL21(DE3) strain was engineered to produce sabinene by introducing a heterologous MVA pathway combining GGPS2 and SabS1 from *Abies grandis* and *Salvia pomifera*, respectively [6]. In this study, ALE was applied to obtain a sabinene-tolerant strain and sabinene production of the evolved strain was tested. Then, whole genome resequencing and transcriptome sequencing of the evolved strain and the parental strain were carried out to identify the genetic basis of improved tolerance. Candidate genes which may be effective in enhancing sabinene tolerance were selected and identified by overexpressing in BL21(DE3). In addition, cell morphology of the evolved strain and the overexpression strains under sabinene stress was monitored by scanning electron microscopy, and sabinene production in the overexpression strains was examined.

## Results and discussion

### Enhancing sabinene tolerance by ALE

**Fig. 2:**
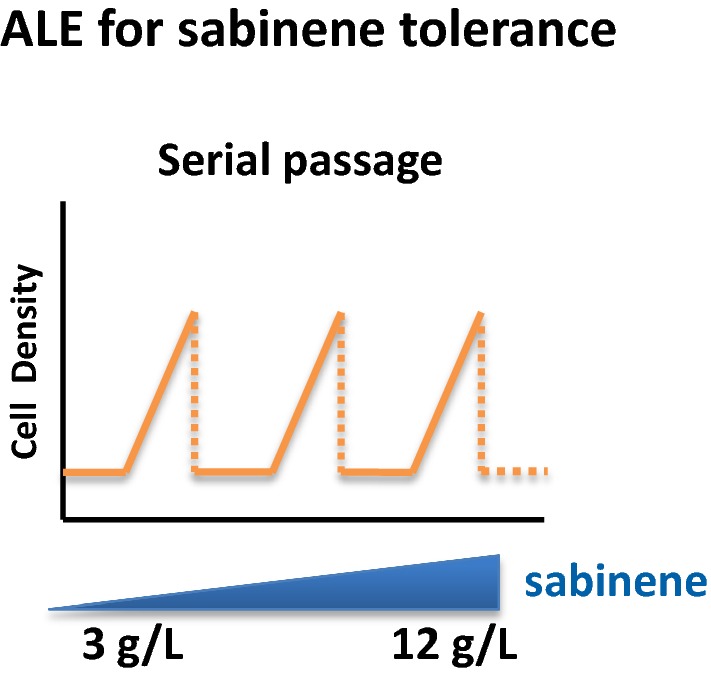
Schematic diagram of the ALE process. Gradually increasing concentrations of sabinene were used as the driving force for the evolution

**Fig. 3:**
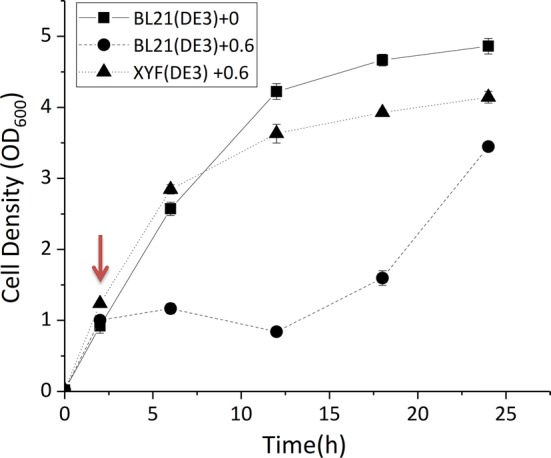
Growth curves of BL21(DE3) and XYF(DE3) under sabinene stress. Squares represent the growth of BL21(DE3) without sabinene stress. Circles and triangles represent the growth of BL21(DE3) and XYF(DE3) with 0.6 g/L sabinene. After 2.5 h of cultivation, sabinene was added to a final concentration of 0.6 g/L. Cell density was monitored for 24 h. The experiments were carried out in triplicates. Error bars represent the standard deviation from the mean

To determine the minimum inhibitory concentration of sabinene to *E. coli* BL21(DE3), growth curve analysis was performed with different concentrations of sabinene. As shown in Additional file 1: Fig. S1, the growth of BL21(DE3) was affected in the present of sabinene and was almost totally inhibited with 3.5 g/L sabinene. Therefore, we chose 3.0 g/L as the initial concentration for ALE. After a series of adaptive evolution with gradually increasing concentrations of sabinene from 3 to 12 g/L, an evolved strain XYF(DE3) was obtained (Fig. 2). XYF(DE3) showed significantly enhanced sabinene tolerance. Growth curve analysis demonstrated that XYF(DE3) showed no growth retardation during the logarithmic growth period and displayed normal growth with 0.6 g/L sabinene, whereas the parental BL21 (DE3) exhibited distinct growth retardation with 0.6 g/L sabinene (Fig. 3). The result proved that ALE is a simple and effective method for enhancing the tolerance of strains to a desired product. This process might involve genetic mutations and systematic transcriptional regulation, and the resulted proteome and metabolome changes, which need to be further investigated.

### The evolved strain showed increased sabinene production

**Fig. 4:**
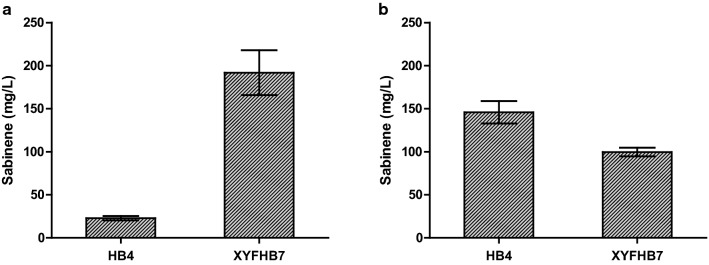
Sabinene production of BL21(DE3) and XYF(DE3). **a** Sabinene production using glucose as a carbon source. **b** Sabinene production using glycerol as a carbon source. HB4, BL21(DE3) harboring pHB7 and pTrcLower. XYFHB7, XYF(DE3) harboring pHB7 and pTrcLower. The experiments were performed in triplicates. Error bars represent the standard deviation from the mean

Sabinene production of the evolved strain was tested. Plasmids pHB7 and pTrcLower were cotransformed into BL21(DE3) and XYF(DE3), respectively, and sabinene-producing strains HB4 and XYFHB7 were obtained (Fig. 1b; Table 1). Shake-flask fermentation of HB4 and XYFHB7 showed noticeable differences in sabinene production between the two strains. Under the condition of glucose as a carbon source, HB4 produced 22.76 mg/L sabinene, while XYFHB7 produced 191.76 mg/L sabinene which was 8.43-fold as much as that of HB4 (Fig. 4a). When using glycerol as the carbon source, a much higher sabinene production (145.92 mg/L) was achieved in HB4 (Fig. 4b), indicating that glucose may inhibit T7 promoter and lead to decrease of sabinene production [27]. However, XYFHB7 produced 99.59 mg/L sabinene which was lower than that of glucose as a carbon source, implying some mutations fixed in XYF(DE3) might lead to lower utilization of glycerol. The above results demonstrated that ALE is a valuable strategy for metabolic engineering of strains for increasing productivity. But to maintain this phenotype in the long run and prevent reverse mutation, we need to understand the mechanism of its tolerance to sabinene.

**Table 1:** Plasmids and strains used in this work

Plasmids/strains	Description	References
Plasmids		
pACYCDuet-1	p15A origin; Cm^R^; P_T7_	Novagen
pHB5	p15A origin; Cm^R^; P_T7_::*GPPS2*-*SabS1*	[6]
pHB7	P15A origin; Cm^R^; P_T7_::*mvaE*-*mvaS*-*GPPS2*-*SabS1*	[6]
pTrcLower	ColE1 origin; Amp^R^; P_trc_::*ERG12*-*ERG8*-*ERG19*-*IDI1*	[6]
pACYC-*ybcK*	p15A origin; Cm^R^; P_T7_::*ybcK*	This work
pACYC-*scpA*	p15A origin; Cm^R^; P_T7_::*scpA*	This work
pACYC-*ygiZ*	p15A origin; Cm^R^; P_T7_::*ygiZ*	This work
pACYC-*ybcK*-*GP* *PS2*-*SabS1*	p15A origin; Cm^R^; P_T7_::*ybcK*-*GPPS2*-*SabS1*	This work
pACYC-*scpA*-*GP* *PS2*-*SabS1*	p15A origin; Cm^R^; P_T7_::*scpA*-*GPPS2*-*SabS1*	This work
pACYC-*ygiZ*-*GPPS2*-*SabS1*	p15A origin; Cm^R^; P_T7_::*ygiZ*-*GPPS2*-*SabS1*	This work
pET28a-*mvaE*-*mvaS*	pBR322 origin; Kan^R^; P_T7_:: *mvaE*-*mvaS*	This work
pGRB	pBR322 origin; Amp^R^; J23119	[63]
pREDCas9	pSC101 origin; Spc^R^; PlacUV5	[63]
Strains		
DH5α	*deoR*, recA1, endA1, hsdR17(rk^−^, mk^+^), phoA, supE44, λ-, thi^−^1, gyrA96, relA1	Invitrogen
BL21(DE3)	*E. coli* B dcm ompT hsdS(rB^−^ mB^−^) gal	Invitrogen
XYF(DE3)	Strain after ALE	This work
Δ*ybcK*	BL21(DE3) *ybck* knock-out strain	This work
XYFHB7	XYF(DE3) harboring pHB7 and pTrcLower	This work
HB4	BL21(DE3) harboring pHB7 and pTrcLower	[6]
WUT1	BL21(DE3) harboring pACYCDuet-1	This work
WUT2	BL21(DE3) harboring pACYC-*ybcK*	This work
WUT3	BL21(DE3) harboring pACYC-*scpA*	This work
WUT4	BL21(DE3) harboring pACYC-*ygiZ*	This work
WUT14	*ΔybcK* harboring pACYCDuet-1	This work
WUT15	BL21(DE3) harboring pHB5, pTrcLower and pET28a-*mvaE*-*mvaS*	This work
WUT16	BL21(DE3) harboring pACYC-*ybcK*-*GPPS2*-*SabS1*, pTrcLower and pET28a-*mvaE*-*mvaS*	This work
WUT17	BL21(DE3) harboring pACYC-*scpA*-*GPPS2*-*SabS1*, pTrcLower and pET28a-*mvaE*-*mvaS*	This work
WUT18	BL21(DE3) harboring pACYC-*ygiZ*-*GPPS2*-*SabS1*, pTrcLower and pET28a-*mvaE*-*mvaS*	This work

### Whole genome resequencing and mutation analysis

To find the mutations fixed in the evolved strain, genomes of XYF(DE3) and parental BL21(DE3) were resequenced with the Illumina HiSeq platform. Compared with the parental strain, 244 single nucleotide variations (SNVs) and 26 insertions and deletions (Indels) were detected in XYF(DE3). A full summary of mutations is given in Additional file 2. The large number of mutations might be caused from the mutation in DNA mismatch repair gene *mutS* (Δ44 bp frameshift deletion), which increased the difficulty of identification of functional mutations. Exonic mutations with high detected frequency were focused on, and among which the frameshift Indels, stop gain SNVs and nonsynonymous SNVs were the most concerns. The encoding gene of the two-component system response regulator RssB, which is involved in general stress response in *E. coli*, was detected to have a Δ1 bp frameshift deletion [28]. Ribonuclease E, which is involved in processing and/or degradation of RNAs and required for induction of the glutamate-dependent acid resistance system, was detected to have a 2 bp frameshift insertion and multiple SNVs in its encoding gene [29]. Superoxide dismutase [Cu–Zn] SodC2, which can protect *E. coli* from oxidative damage, was detected to have a 1 bp frameshift insertion in its encoding gene [30]. The lauroyl-Kdo2-lipid IVA myristoyltransferase (*lpxM*), which is associated with lipid A modification and required for bacterial survival and persistence in various environments by optimizing bacterial membrane structure and/or integrity, was detected to have a stop gain mutation [31].

Other noteworthy SNVs-containing genes can be classified according to their annotations into at least 7 groups: (1) polysaccharide/lipid metabolism-related genes which may play roles in biofilm, cell wall, or membrane formation and stability [32], such as polysaccharide export protein *gfcE* [33], 3-oxoacyl-ACP reductase *fabG* [34], GntR family transcriptional regulator *fadR* [35], and putative aminodeoxychorismate lyase *yceG* [36, 37]. (2) Kinase and outer-membrane receptor genes which may be required for signaling transduction and response to oxidative stress or exogenous toxicant, such as the two-component system sensor histidine kinase *pmrB* [38–40] and iron uptake receptor *fhuE* [41]. (3) Nucleic acid biosynthesis, processing, or degradation-related genes which may be involved in repair or degradation of damaged DNA/RNA during stress, such as ribonuclease E *rne* and DNA polymerase III subunit delta’ *holB*. (4) Carbohydrate metabolism-related genes which may confer tolerance through regulation of carbon source metabolism and energy conversion, such as MFS transporter *galP*, PTS system fructose-specific IIB/IIC component *frvB*, and 2-hydroxy-3-oxopropionate reductase *glxR*. (5) Aldehyde dehydrogenase genes which play a major role in the detoxification processes of aldehydes generated during oxidative stress, such as the nonspecific aldehyde dehydrogenase *puuC* [42]. (6) Multidrug efflux transporter and related regulator genes which regulate tolerance by exporting toxic organic solvents to the external medium, such as multidrug efflux transporter EmrAB transcriptional repressor *mprA* (MarR family) [43] and multidrug efflux pump *arcB* [44]. (7) Cell division-related genes which may be associated with maintaining of normal cell morphology, such as *zapE* [45]. The above genes are ideal candidates for mutation reconstruction analysis, among which deletion of *fadR* and *marR* was reported to have increased organic solvent tolerance of *E. coli* in a previous study [35]. In addition, a lot of flagellar structure or assembly-related genes were found to have multiple mutations. Because BL21(DE3) has no flagellum, whether these mutations were responsible for the enhanced sabinene tolerance of XYF(DE3) need further investigation.

### Transcriptome analysis reveals important metabolic pathways and genes involved in sabinene tolerance

**Fig. 5:**
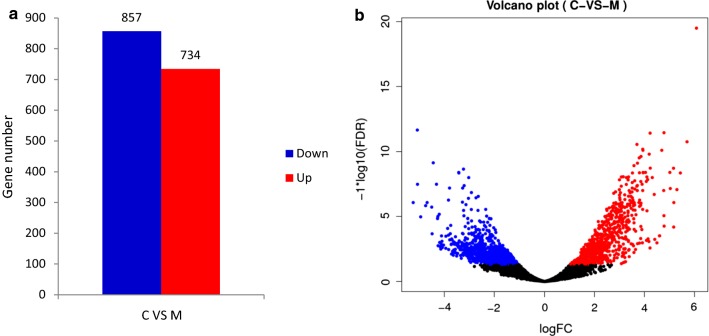
Differentially expressed genes through transcriptome analysis. **a** The total number of the up-regulated and down-regulated genes between the control strain C and the evolved strain M. **b** The volcano plot of the differential genes between the control strain C and the evolved strain M. Red represents up-regulated genes, and blue represents down-regulated genes

**Fig. 6:**
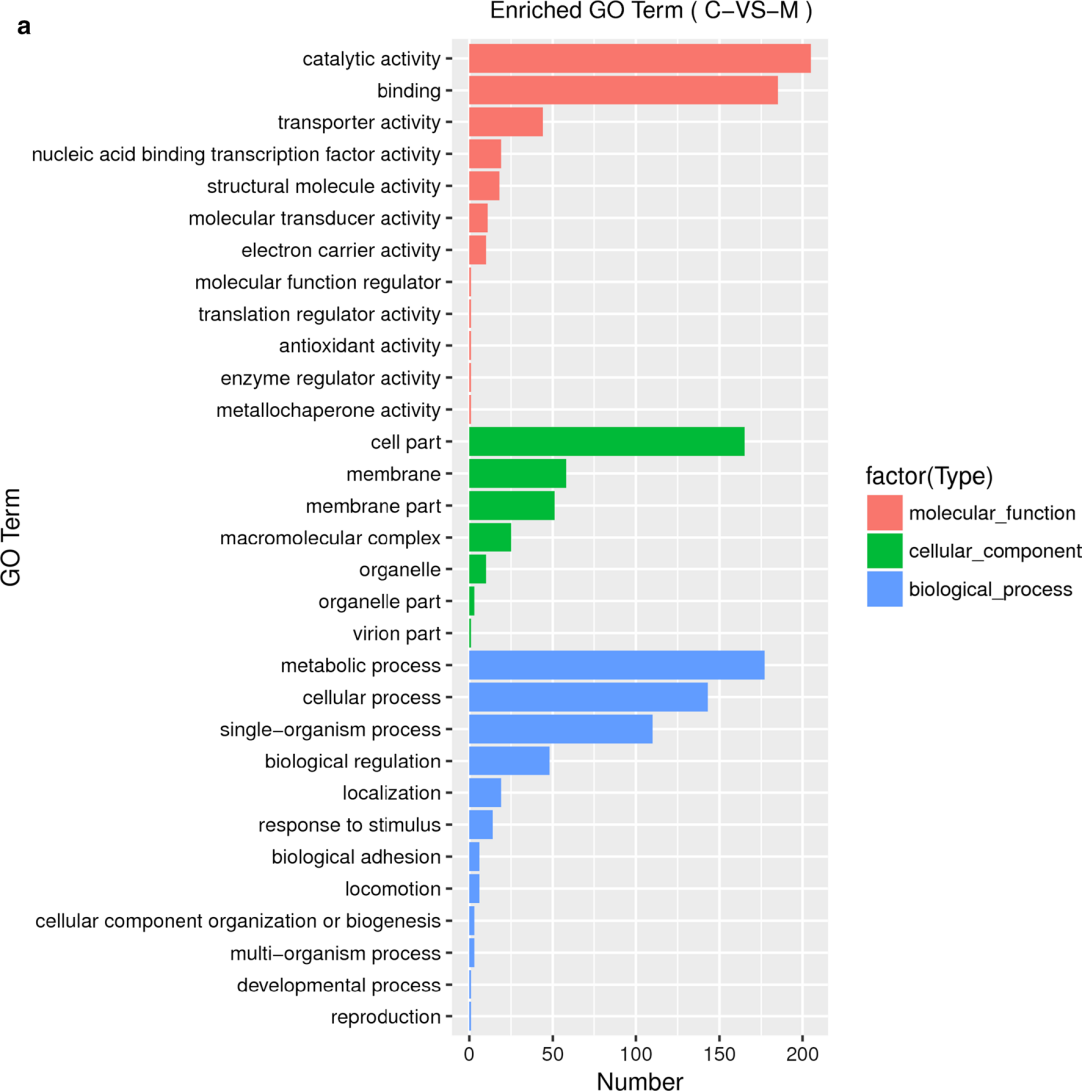

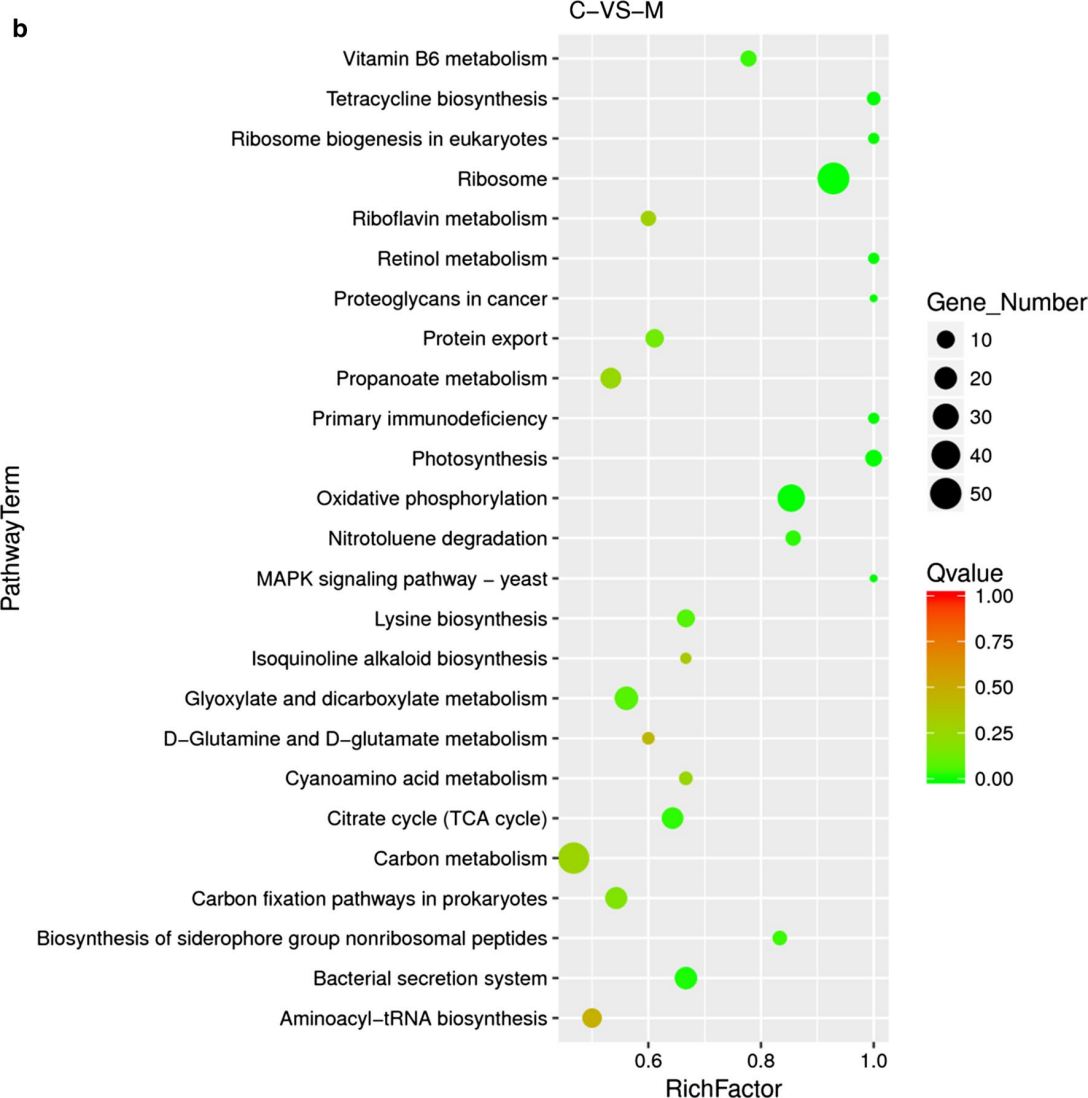
GO and KEGG enrichment analyses of the differentially expressed genes. **a** Histogram of GO enrichment analysis. The vertical coordinate shows the enriched GO terms, and the horizontal coordinate represents the number of differentially expressed genes in the term. Different colors have been used to distinguish molecular function, cellular component, and biological process; **b** Scatter plot of the KEGG enrichment analysis of differentially expressed genes. The vertical coordinate shows the pathway names; the horizontal coordinate shows the Rich Factor; the dot size indicates the number of differentially expressed genes in the pathway; and the color of the dot corresponds to different Q value ranges

To decipher the molecular mechanism of tolerance improvement of the evolved strain at transcriptional level, RNA sequencing and transcriptome analysis of BL21(DE3) and XYF(DE3) grown under 0.5 mg/L sabinene were also performed. 734 up-regulated genes and 857 down-regulated genes were identified in the evolved strain (marked as M) comparing with the parental strain (marked as C) through differential expression analysis (Fig. 5). Gene Ontology (GO) enrichment analysis was carried out to classify the differentially expressed genes. In total, 962 genes were annotated with GO terms. 313, 217, and 317 genes were annotated with molecular function, cellular component, and biological process, respectively **(**Fig. 6a**)**. In the molecular functional category, 67.1% and 53.3% are related to catalytic activity and protein binding. Within the cellular component category, 79.7% and 39.6% of the genes are associated with cell part and membrane. In the biological process category, 55.5% of the genes are associated with metabolic process while 42.9% are related to cellular process.

In terms of Kyoto Encyclopedia of Genes and Genomes (KEGG) enrichment analysis, 2905 genes were annotated within KEGG pathways. The significantly regulated pathways are listed in Fig. 6b. 52 differentially expressed genes are enriched in ribosome pathway, indicating ribosome regulation is a strategy to overcome the sabinene stress which may lead to ribosome disruption and inhibition of protein synthesis, similar as the response of *E. coli* to antibiotics stress [46–48]. Vitamin B6 metabolism, photosynthesis, and oxidative phosphorylation pathways also show highly reliable regulation, implying that these pathways may be involved in resistance against oxidative stress caused by sabinene treatment and maintain internal redox environment balance [49–51]. In addition, expression of genes associated with fatty acid synthesis pathways was down-regulated, whereas those associated with fatty acid degradation pathways were up-regulated (Additional file 1: Fig. S2). Expression of some genes associated with biofilm formation was up-regulated, which was consistent with the genome resequencing analysis that biofilm formation might be important in improving sabinene tolerance. Expressions of mismatch repair-related genes and MEP pathway genes were down-regulated, implying these pathways may be also involved in response to sabinene stress.

The top 100 up-regulated genes were analyzed (Additional file 1: Table S3). By comparing the data with the UniProt and KEGG databases, we found that 22% of the genes encode uncharacterized proteins. Others encode proteins involved in various biological processes or metabolic pathways. According to the results of genome resequencing and transcriptome analyses, 12 candidate genes encoding proteins involved in biofilm formation, stress response, carbohydrate metabolism or signal transduction were selected for reverse engineering (Table 2) [52–55].

**Table 2:** Candidate genes selected for reverse engineering

GeneSymbol	Length	log2FoldChange	*p* value adjusted	Description
*ymgJ*	186	5.157986885	1.94E−09	General stress-inducible protein YmgJ
*ygiZ*	333	4.775909937	3.54E−12	Inner membrane protein YgiZ
*atoD*	663	4.231411852	1.89E−09	Acetate CoA-transferase subunit alpha AtoD
*ybcK*	1527	3.939606713	7.99E−11	DLP12 prophage family protein YbcK
*kilR*	222	3.931475332	1.00E−07	Killing protein KilR
*torY*	1101	3.907124887	3.29E−08	Cytochrome c-type protein TorY
*ybcV*	411	3.510490058	6.46E−08	DLP12 prophage family protein YbcV
*ydiM*	1215	3.217260596	4.96E−07	Putative exporter YdiM
*bglH*	1617	3.151768069	8.75E−09	Cryptic outer membrane porin BglH
*elaD*	1212	3.070684375	4.65E−08	Protease ElaD
*bglF*	1878	3.039815419	1.48E−07	Beta-glucoside-specific PTS enzyme II/BglG kinase/BglG phosphatase BglF
*scpA*	2145	1.424758051	1.40E−02	Methylmalonyl-CoA mutase ScpA

### Validation of the sabinene tolerance-related genes

**Fig. 7:**
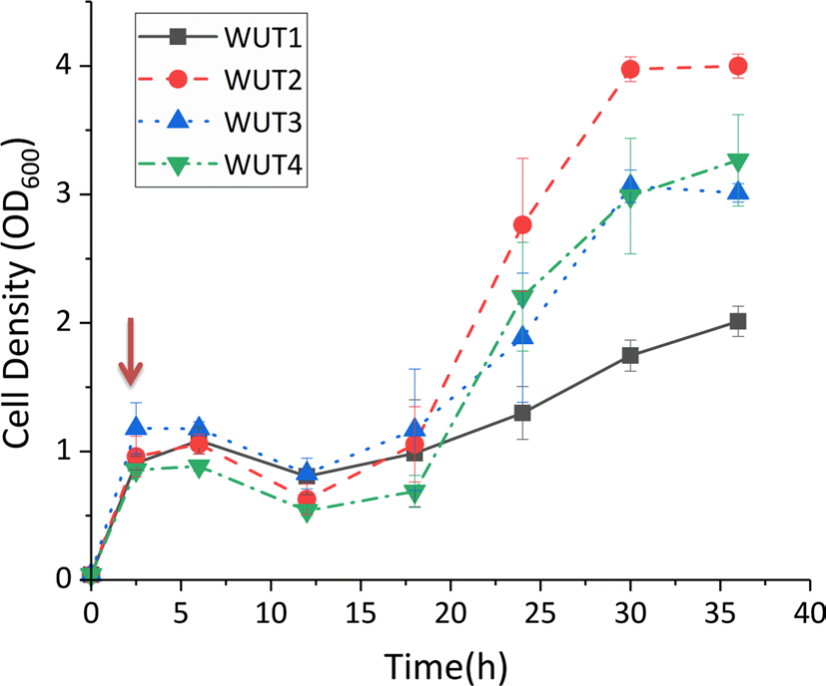
Growth curves of reverse-engineered strains under sabinene stress. Squares with solid lines indicate strain WUT1, expressing empty plasmid pACYCDuet-1. Circles with dashed lines indicate strain WUT2, overexpressing gene *ybcK*. Triangles with dashed lines indicate strain WUT3, overexpressing gene *scpA*. Inverted triangles with dashed lines indicate strain WUT4, overexpressing gene *ygiZ*. The experiments were carried out in triplicates. Error bars represent the standard deviation from the mean

Reverse engineering was carried out to verify if the 12 candidate genes were involved in regulation of sabinene tolerance in *E. coli*. The genes were cloned into vector pACYCDuet-1 and then expressed in BL21 (DE3), respectively (Table 1; Additional file 1: Table S1). As shown in Fig. 7 and Additional file 1: Fig. S4, overexpression of three candidate genes (*ybcK, scpA* and *ygiZ*) could significantly improve growth of the engineered strains under 0.6 g/L sabinene. After 30 h of cultivation, the OD_600_ of WUT1 was 1.746, whereas OD_600_ of WUT2, WUT3, and WUT4 reached 3.975, 3.063, and 2.987, respectively. WUT2 showed the greatest OD_600_ difference with WUT1 after 30 h of cultivation. In terms of biomass, the tolerance of strains overexpressing *ybcK* (Gene ID: 945166)*, scpA* (Gene ID: 945576), and *ygiZ* (Gene ID: 946450) increased by 127.7%, 75.4%, and 71.1% compared with the control strain at 30 h, respectively (Fig. 7). Moreover, we generated a *ybcK* knock-out mutant (*ΔybcK*). As shown in Additional file 1: Fig. S5, Δ*ybcK* harboring the control plasmid (WUT14) showed significant reduced sabinene tolerance, suggesting that *ybcK* gene plays important roles in response to sabinene stress in *E. coli*. However, the retarded growth of the engineered strains in the first dozen hours indicated that other genes may be involved in sabinene stress response.

*ybcK* is a function-unknown gene that belongs to the DLP12 (defective Lambda prophage) family. DLP12 family genes were obtained from phages during long-term predation by bacteriophages to promote bacterial survival and were demonstrated to play important roles in biofilm formation, stress response, and cell wall maintenance [56]. *scpA* encodes the methylmalonyl-CoA mutase which is involved in many metabolic processes. It catalyzes the reversible stereotactic conversion of methylmalonyl-CoA to succinyl-CoA, which accelerates the glyoxylic acid cycle and can enhance the production of succinyl-CoA. Succinyl-CoA, which is involved in the energy metabolism of cells, promotes the TCA cycle and improves cell growth. Simultaneously, the consumption of malonyl-CoA shifts the propionic acid balance, which can prevent the accumulation of propionic acid and is beneficial for cell growth. We hypothesis that *scpA* may enhance the sabinene tolerance of the engineered strains through regulation of carbon and energy metabolism. *ygiZ* encodes an uncharacterized inner membrane protein. This gene was demonstrated to be regulated by the BglJ-RcsB transcriptional activator which was involved in motility, biofilm formation, and various stress responses [57]. Our results indicate *ybcK* and *ygiZ* may enhance the sabinene tolerance of the engineered strains through regulation of cell wall status and/or biofilm formation. However, the detailed function of *ybcK*, *scpA*, and *ygiZ* in improving sabinene tolerance need to be further investigated.

### The evolved strain displays normal morphology under sabinene stress

**Fig. 8:**
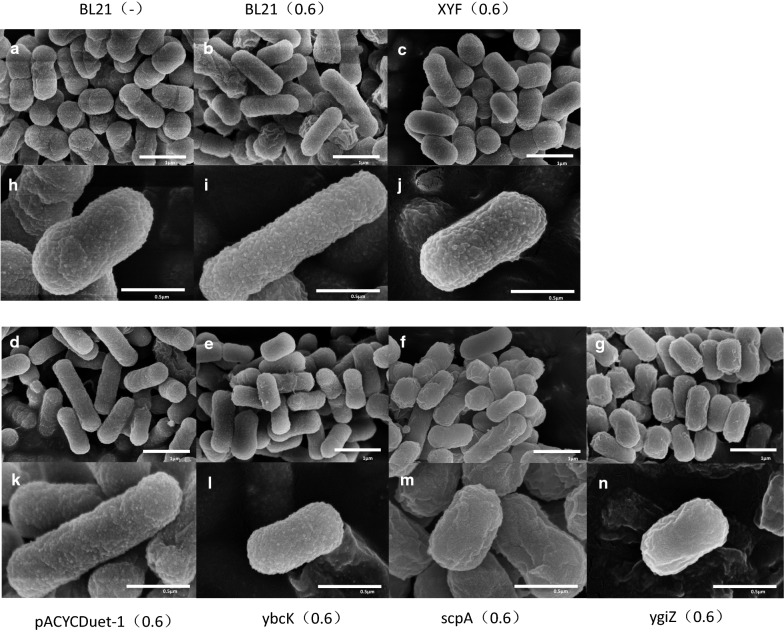
Electron micrographs of different strains grown with or without sabinene treatment. **a** Cell morphology of BL21(DE3) grown without sabinene; **b** cell morphology of BL21(DE3) grown with 0.6 g/L sabinene; **c** cell morphology of XYF(DE3) grown with 0.6 g/L sabinene; **d**–**g** cell morphology of WUT1, WUT2, WUT3, and WUT4 grown with 0.6 g/L sabinene, respectively. **h**–**n** (magnification of ×80,000) are enlarged images of **a**–**g** (magnification of ×30,000), respectively

In addition to reverse engineering, we also evaluated cell morphology under sabinene stress. Samples of BL21(DE3), XYF(DE3) and the reverse engineered strains were prepared and observed by electron microscopy. BL21(DE3) showed increased cell length under sabinene stress, reaching approximately 1.4 µm, whereas the evolved strain XYF(DE3) displayed normal morphology (0.9 µm) similar to BL21(DE3) cells grown without sabinene (Fig. 8; Additional file 1: Fig. S6). Genetically engineered strains or strains grown under various stresses often showed retarded growth and increased cell length [58, 59]. Our results proved sabinene stress can also cause growth retardation and morphology change, and lead to decreased cell density (OD_600_) of *E. coli*. In contrast, the evolved XYF(DE3) exhibited significantly improved adaptation to sabinene stress and grew in a manner similar to that of BL21(DE3) grown in the absence of sabinene which possibly due to the regulation of multiple mutant genes and the resulted variations of transcriptome, proteome, and metabolome.

### Overexpression of *ybcK*, *scpA*, or *ygiZ* rescue *E. coli* morphology under sabinene stress

Cell morphologies of strains expressing *ybcK* (WUT2), *scpA* (WUT3), or *ygiZ* (WUT4) were examined. Under sabinene stress, cells of WUT2, WUT3, and WUT4 showed similar shape and cell length to that of BL21(DE3) grown without sabinene (Fig. 8; Additional file 1: Fig. S6), suggesting that overexpression of *ybcK*, *scpA*, or *ygiZ* gene could at least partially restore the cell morphology of engineered strains grown under sabinene stress. The result also proved the improvement of sabinene tolerance by overexpressing *ybcK*, *scpA*, or *ygiZ*, which is in agreement with our results that overexpression of *ybcK*, *scpA*, or *ygiZ* could improve the growth of the engineered strains to higher cell densities than that of the control strain. The above results demonstrated that *ybcK*, *scpA*, and *ygiZ* play important roles in response to sabinene stress in *E. coli*.

### Overexpression of *scpA* or *ygiZ* can increase sabinene production

As sabinene tolerance was improved and cell morphologies were rescued under sabinene stress in *ybcK*, *scpA*, or *ygiZ* overexpression strains, we wonder if sabinene production can be increased in these strains. Sabinene biosynthetic pathway was introduced into WUT1, − 2, − 3, − 4, respectively, and the corresponding strains WUT15, − 16, − 17, − 18 were obtained. As shown in Additional file 1: Fig. S7, sabinene production in *scpA* overexpression strain (WUT 17) was about 2 times as much as that of the control strain when using glucose as the carbon source, while sabinene production in *scpA* or *ygiZ* overexpression strains (WUT17 and WUT18) was 1.94-fold and 2.45-fold as much as that of the control strain when using glycerol as the carbon source, respectively. In contrast, *ybcK* overexpression strain (WUT16) showed decreased sabinene production in both media. The above results suggested that host tolerance is one of the limiting factors for sabinene production in *E. coli* and other factors, such as carbon source, are also involved.

## Conclusions

In this study, we used sabinene as the selective pressure to drive the evolution of *E. coli* BL21(DE3) and obtained an evolved strain XYF(DE3). The improved sabinene tolerance and production of XYF(DE3) demonstrated that ALE is an effective strategy for metabolic engineering. Whole genome resequencing analysis revealed that XYF(DE3) is a hypermutator with 244 SNVs and 26 Indels. Transcriptome analysis of the evolved strain and the parental strain suggested that ribosome regulation, biofilm formation, and redox homeostasis-related pathways might be important for resistance against sabinene stress. Twelve up-regulated genes were selected for reverse engineering and three of them, *ybcK*, *scpA*, and *ygiZ*, were identified to be important for sabinene tolerance improvement. Sabinene production in *scpA* or *ygiZ* overexpression strains was increased. Furthermore, scanning electron microscopy demonstrated that the evolved strain and the strains overexpressing *ybcK*, *scpA*, or *ygiZ* showed normal cell morphology under sabinene stress. The study provided valuable information for design of rational strategies to optimize the microbial biosynthesis system of sabinene or even other terpenoids. The molecular mechanism underlying the improved tolerance and the detailed function of each gene will be investigated in future work.

## Materials and methods

### Strains, plasmids, and media

*E. coli* BL21(DE3) was chosen as the background strain for this work. Plasmids pHB7 harboring *acetyl*-*CoA acetyltransferase/HMG*-*CoA reductase* (*mvaE*), *HMG*-*CoA synthase* (*mvaS*), *geranyl diphosphate synthase* (*GPPS2*) and *sabinene synthase* (*SabS1*) and pTrcLower harboring *mevalonate kinase* (*ERG12*), *phosphomevalonate kinase* (*ERG8*), *mevalonate pyrophosphate decarboxylase* (*ERG19*), and *IPP isomerase* (*IDI*) were used for sabinene biosynthesis [6]. All the strains and plasmids used in this study are listed in Table 1 and Additional file 1: Table S1. Strains were grown routinely in Luria–Bertani (LB) broth (supplemented with antibiotics if necessary). For shake-flask fermentation, a modified M9 medium described by a previous work was used [6]. DNA polymerase, restriction enzymes and DNA ligase were purchased from Thermo Fisher Scientific.

### Adaptive laboratory evolution (ALE) for sabinene tolerance

The minimum inhibitory concentration of sabinene to *E. coli* BL21(DE3) was tested first. 500 μL seed culture of BL21(DE3) was inoculated into saline bottles containing 50 mL LB medium. Then, sabinene was added into the medium to make final concentrations of 0, 0.5, 1.0, 1.5, 2.0, 2.5, 3.0, 3.5, and 4.0 g/L, respectively, and the bottles were sealed. After 24 h of cultivation in a shaker at 37 °C, OD_600_ of the cultures was measured. ALE was applied to BL21(DE3) in LB medium. 500 μL seed culture was inoculated into saline bottles containing 50 mL LB medium supplemented with 3 g/L sabinene, and cultivated in a shaker at 37 °C for 24 h. Then, culture (1% inoculation scale) was transferred to fresh medium with the same sabinene concentration and cultivated at 37 °C for another 24 h. Subsequently, sabinene concentration was gradually increased from 3 to 12 g/L in 0.6 g/L steps during the ALE process. The strains were cultivated 2 times (24 h for each propagation cycle) under each concentration. The strain that could tolerate 12 g/L sabinene was cultured for 24 h for three times under the same concentration to obtain a genetically stable generation. Colonies were isolated through plate streaking and the best sabinene-tolerant evolved strain was named XYF(DE3) and stored as glycerol stock at − 80 °C.

Growth curves of BL21(DE3) and XYF(DE3) were measured under 0.6 g/L sabinene. The strains were cultivated as mentioned above and 0.6 g/L sabinene was added into the medium after 2.5 h of cultivation. Cultures were further incubated at 37 °C for 21.5 h and OD600 was measured periodically.

### Shake-flask fermentation and sabinene quantification

Shake-flask fermentation was carried out in triplicate according to the method described by Zhang [6]. Different strains were inoculated in 50 ml of fermentation medium containing the corresponding antibiotics and cultivated in a shaker at 37 °C 200 rpm. When OD600 of the bacterial culture reached 0.6–0.9, cells were induced by IPTG at a final concentration of 0.1 mM at 30 °C 200 rpm. After 24 h of fermentation, 5 mL cyclohexane was injected into each bottle and the culture was continued incubated in the shaker at 30 °C 200 rpm for additional 16 h. Culture was centrifuged and 1 μL upper organic phase was analyzed by gas chromatography (GC) using an Agilent 7890B GC system equipped with a DB-5MS column. The GC conditions were as follows: the oven temperature was initially held at 50 °C for 0.5 min, increased at 10 °C/min to 120 °C and held for 0.5 min, and then increased at 20 °C/min to 180 °C and held for 1 min. The temperatures of injector and detector were held at 250 °C and 260 °C, respectively (Additional file 1: Fig. S3). The peak area was converted into sabinene concentration in comparison with a standard curve plotted with a set of known concentrations of commercial sabinene bought from Sigma-Aldrich.

### Genome resequencing analysis

Two clones from each of the evolved strain XYF(DE3) and the parental strain BL21(DE3) were genome sequenced through Illumina HiSeq. Genome DNA (gDNA) extraction, gDNA treatment and sequencing, and data analyses were all completed by GENEWIZ Suzhou, Inc. Quality of raw data was optimized using cutadapt (v1.9.1) to eliminate adaptor sequences and low-quality sequences. The optimized data were analyzed and assembled using Velvet (v1.2.10), SSPACE (v3.0) and GapFiller (v1-10). Genes and non-coding RNA were predicted by Prodigal (v3.02) and Rfam (v12.0), respectively. Genes’ annotation information was obtained based on blast to GO, KEGG, NR (Non-Redundant Protein Database), COG (Cluster of Orthologous Groups of proteins) and other databases. SNVs and Indels were detected in XYF(DE3) compared with BL21(DE3) using Samtools (v1.1) and GATK UnifiedGenotyper.

### RNA extraction and transcriptome analysis

Each clone of BL21(DE3) and XYF(DE3) was incubated in 5 mL LB medium containing 0.5 g/L sabinese and cultivated in a shaker at 37 °C overnight. Cells were collected by centrifugation and two samples were prepared from each strain, naming C1, C2 and M1, M2, respectively. Total RNA from the *E. coli* cells was extracted using TRIzol reagent. The quality and concentration of the extracted RNAs were examined by Agilent 2100 Bioanalyzer. 1 μg total RNA with RIN value above 7 was used for the following process. mRNA library preparation, RNA sequencing (Illumina HiSeq), and data processing were accomplished by GENEWIZ Beijing, Inc.

Differential expression analysis between the parental strain (C, merging C1 and C2 data) and the evolved strain (M, merging M1 and M2 data) was completed by DESeq2 package [60]. *p* value was calculated using Wald test and adjusted by Benjamini–Hochberg procedure for controlling the false-discovery rate (FDR) [61, 62]. Adjusted *p* value < 0.05 and log2 Fold Change ≥ 2 were determined to detect differentially expressed genes. The differentially expressed genes were arranged according to the log2 Fold Change in descending order and the top 100 up-regulated genes are listed in Additional file 1: Table S3. Twelve candidate genes were selected for further reverse engineering (Table 2).

Statistics analysis and cluster analysis, including GO and KEGG enrichment, were performed to the differentially expressed genes. GO analysis first maps all the differentially expressed genes to GO database, and then calculates the number of differential genes in each term. The GO terms that are enriched with differential genes were determined using hypergeometric test against the genomic background. The equation is as below:

$$p = 1 - \sum\limits_{i = 0}^{m - 1} {\frac{{\left( \begin{aligned} & M \\ & i \\ \end{aligned} \right)\left( \begin{aligned} & N - M \\ & n - i \\ \end{aligned} \right)}}{{\left( \begin{aligned} & N \\ & n \\ \end{aligned} \right)}}} ,$$

where *N* is the number of all the genes included in the GO annotation; *n* the number of differentially expressed genes in *N*; *M* the number of genes annotated for a particular GO term; and *m* the number of differential genes in each particular GO term. The calculated *p* value was corrected by the Bonferroni method. Differentially expressed genes are considered significantly enriched with GO terms if the corrected *p* value is ≤ 0.05. Histogram of GO enrichment was drawn according to the instructions on its website (http://geneontology.org/docs/go-enrichment-analysis/, Accessed 10 Sep 2019).

Pathway analysis is based on KEGG pathway units and used a hypergeometric test to find pathways of the differentially expressed genes that are significantly enriched against the transcriptome background. Below is the formula:

$$p = 1 - \sum\limits_{i = 0}^{m - 1} {\frac{{\left( \begin{aligned} & M \\ & i \\ \end{aligned} \right)\left( \begin{aligned} & N - M \\ & n - i \\ \end{aligned} \right)}}{{\left( \begin{aligned} & N \\ & n \\ \end{aligned} \right)}}} ,$$

where *N* is the number of genes with pathway annotations; *n* the number of differentially expressed genes in *N*; *M* the number of genes annotated for a particular pathway in all genes; and *m* the number of differentially expressed genes annotated for that pathway. The calculated *p* value was corrected by the Bonferroni method. Differentially expressed genes are considered significantly enriched with KEGG pathways if the corrected *p* value is ≤ 0.05. Scatter plot of KEGG enrichment was drawn by the ggplot2 package on R platform.

### Reverse engineering for candidate genes

After candidate genes were selected, plasmids’ construction and overexpression of the selected genes were carried out. *E. coli* BL21(DE3) genomic DNA was used as a template for amplification of candidate genes. Gene fragments were inserted into pACYCDuet-1 through digestion and ligation, and primers as well as restriction enzymes used are listed in Additional file 1: Table S2. The constructed plasmids were transformed into BL21(DE3). Single colonies of the engineered strains were incubated overnight in 5 mL LB medium containing Cm at 37 °C. Then, seed culture with the equivalent biomass was inoculated into 50 mL LB medium in saline bottles and cultivated in a shaker at 37 °C. After 2.5 h of cultivation (OD_600_ reached 0.6–0.9), IPTG and sabinene were added to final concentrations of 0.25 mM and 0.6 g/L, respectively. The culture was further incubated at 30 °C for 33.5 h and OD_600_ was measured periodically.

An *ybcK* knock-out mutant (*ΔybcK*) was generated through CRISPR–Cas9 technique according to the method described by Li et al. [63]. Primers used for constructing guide RNA (gRNA) plasmid and donor DNA are listed in Additional file 1: Table S2. *ΔybcK* mutant was transformed with empty plasmid pACYCDuet-1 and used for sabinene tolerance analysis.

To examine sabinene production in strains WUT2, WUT3, and WUT4, plasmids pACYC-*ybcK/scpA/ygiZ*-*GPPS2*-*SabS1* and pET28a-*mvaE*-*mvaS* were constructed using NEBuilder HiFi DNA Assembly. *ybcK/scpA/ygiZ* fragments were amplified from pACYC-*ybcK/scpA/ygiZ* and inserted into pHB5, respectively. *mvaE*-*mvaS* fragment was amplified from pHB7 and inserted into pET28a. Primers used are listed in Additional file 1: Table S2.

### Morphological observation by scanning electron microscopy (SEM)

Morphology of strains treated with or without sabinene was observed by SEM (Table 3). 500 μL seed culture was inoculated into 50 mL LB medium and cultivated at 37 °C. After 2.5 h of growth, 0.6 g/L sabinene, 0.25 mM IPTG, and 34 μg/L Cm (if necessary) were added and the culture was incubated at 30 °C for an additional 30 h. Sample preparation was performed according to the method mentioned by Chen [64]. In brief, cells were pelleted and washed with PBS three times, and then fixed with 2.5% v/v glutaraldehyde and post-fixed with 1% v/v osmium tetroxide. After dehydration in a series of ethanol solutions (30%, 50%, 70%, 90% and 100%), cells were suspended in the mixture of ethanol and tert-butyl alcohol, submerged in 100% tert-butyl alcohol and then dried on critical point of tert-butyl alcohol. Samples were sputter-coated with platinum and examined using a HITACHI S-4800 scanning electron microscope.

**Table 3:** Strains and conditions used for SEM observation

Strains	Sabinene (g/L)	Cm (mg/L)	IPTG (mM)
BL21(DE3)	0	0	0
BL21(DE3)	0.6	0	0
XYF(DE3)	0.6	0	0
WUT1	0.6	34	0.25
WUT2	0.6	34	0.25
WUT3	0.6	34	0.25
WUT4	0.6	34	0.25

## Supplementary information

**Additional file 1: Fig. S1.** Cell density of *E. coli* BL21 (DE3) under different concentrations of sabinene stress. **Fig. S2.** Hierarchical clustering. **Fig. S3.** Quantification of sabinene production by Gas Chromatography. **Fig. S4.** Cell density of all the reverse-engineered strains grown after 24 h of cultivation with 0.6 g/L sabinene. **Fig. S5.** Growth curves of reverse-engineered strains under sabinene stress. **Fig. S6.** Cell length statistics of different strains. **Fig. S7.** Sabinene production of *ybcK*, *scpA* or *ygiZ* overexpression strains. **Table S1.** Other reverse-engineered strains. **Table S2.** Primers used for construction of the reverse-engineered strains and*ΔybcK.***Table S3.** The top 100 up-regulated genes.

**Additional file 2:** Summary of mutations.

## Data Availability

All data generated during this study are included in this manuscript and in Additional files 1, 2.

## Abbreviations

*E. coli*: *Escherichia coli*
ALE: Adaptive laboratory evolution
SEM: Scanning electron microscopy
GO: Gene ontology
KEGG: Kyoto Encyclopedia of Genes and Genomes
MVA: Mevalonate
MEP: Methylerythritol 4-phosphate
