# Supplementary material for: Improvement of sabinene tolerance of Escherichia coli using adaptive laboratory evolution and omics technologies
Source: Biotechnol Biofuels. 2020 Apr 24;13:79. doi: 10.1186/s13068-020-01715-x (PMC7181518; doi:10.1186/s13068-020-01715-x)
Supplement: Supplementary file 2 — Additional file 2: Summary of mutations. [file 13068_2020_1715_MOESM2_ESM.doc]

**Supplementary data**


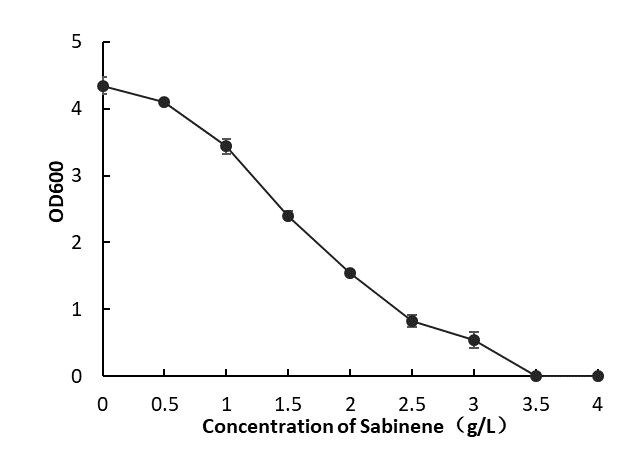


**Fig. S1** Cell density of *E. coli* BL21(DE3) grown with different concentrations of sabinene. The figure shows the cell density curve of BL21(DE3) after 24 h of cultivation under different concentrations of sabinene stress. The growth of BL21(DE3) was completely inhibited under 3.5 g/L sabinene. The experiments were carried out in triplicate. Error bars represent the standard deviation from the mean.

**
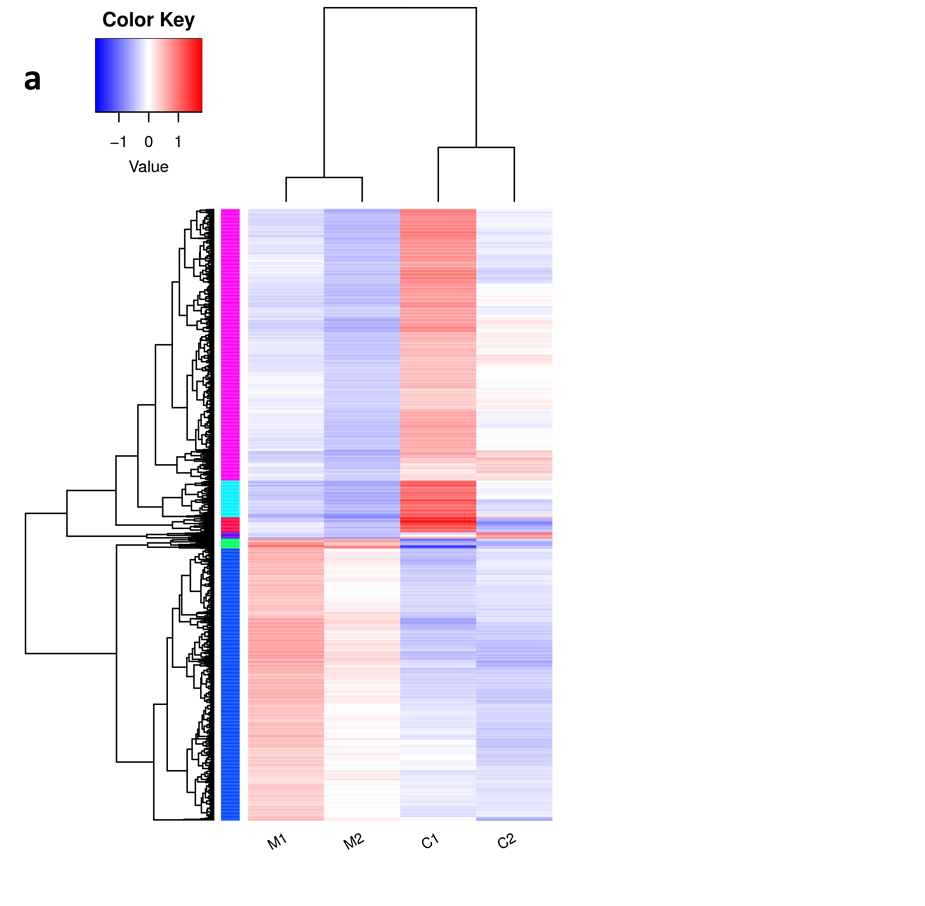
**

**b**


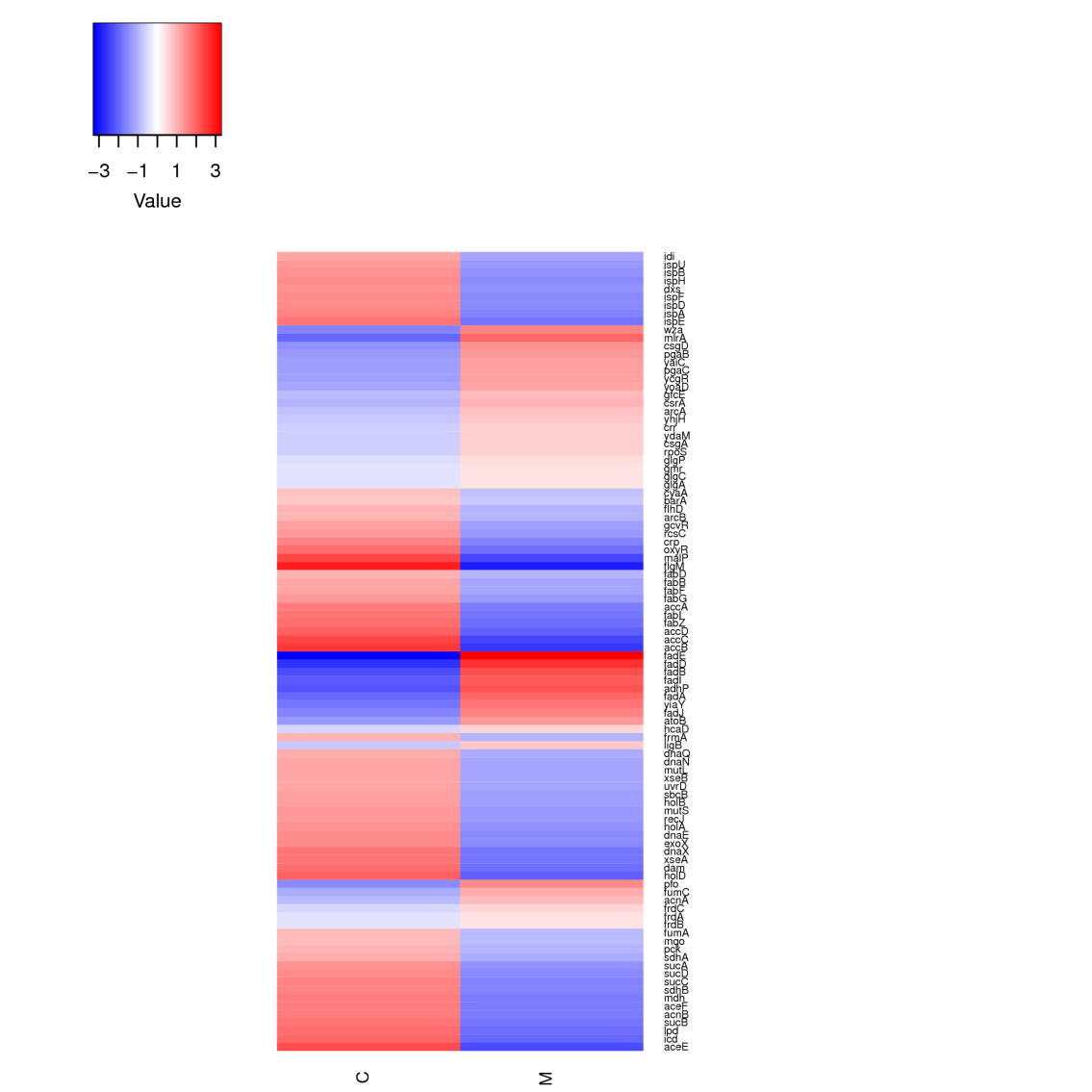


Terpenoid backbone biosynthesis(ko00900)

Biofilm formation (ko02026)

Fatty acid biosynthesis (ko00061)

Fatty acid degradation (ko00071)

Mismatch repair (ko03430)


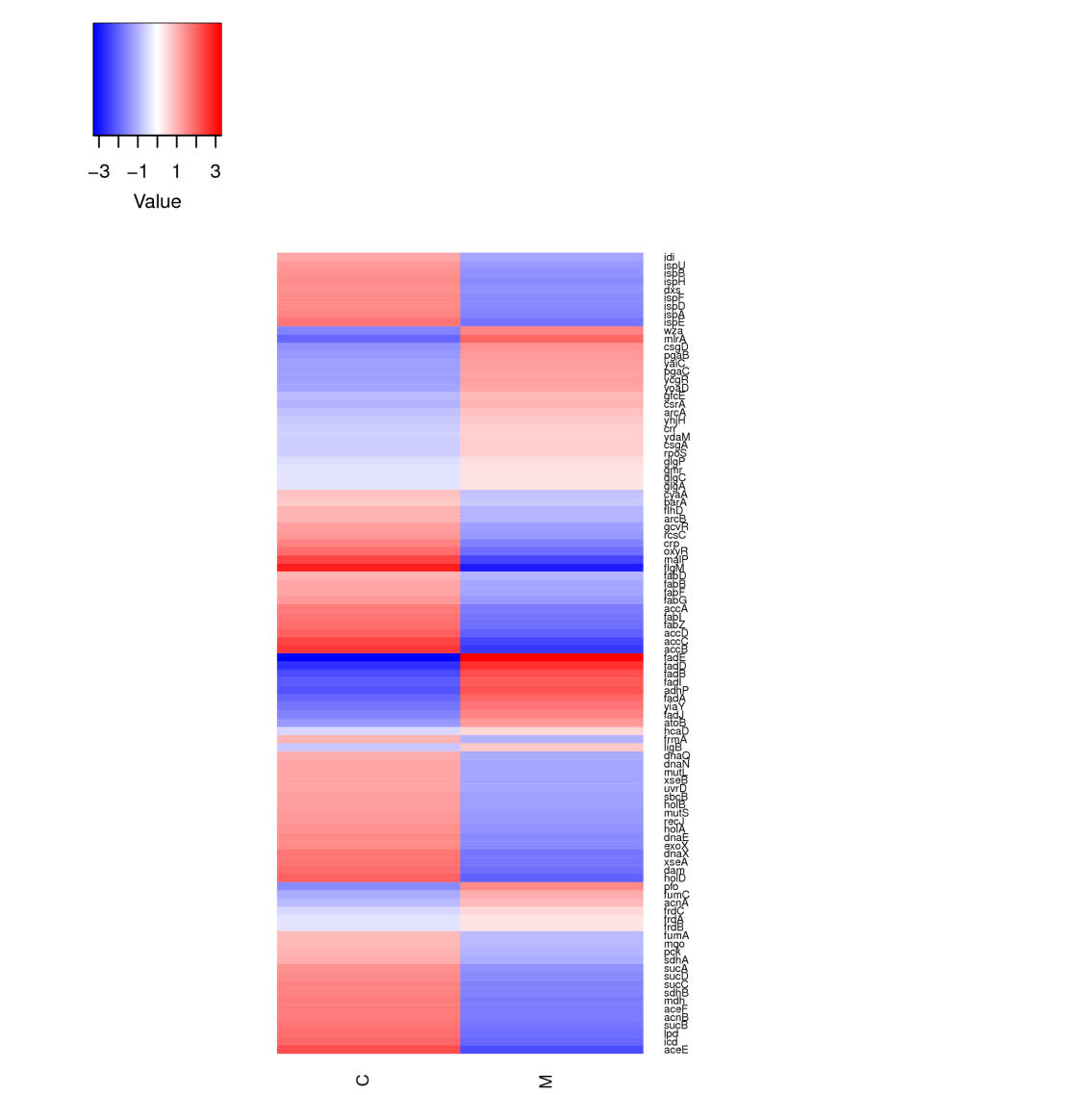


**Fig. S2** Hierarchical clustering. **a** Differential expression gene cluster analysis. Log10 (RPKM+1) were used for clustering, high expression genes are in red and low expression are in blue. Color ranges from blue to red, indicating a higher amount of gene expression. **b** Cluster analysis of significant differential pathway genes. Red bars represent a relative up-regulation of the gene, while blue represents a relative down-regulation of the gene. The figure shows five paths, terpenoids backbone biosynthesis, biofilm formation, fatty acid biosynthesis, fatty acid degradation and mismatch repair. The differentially expressed genes in each pathway are arranged according to the Log2 Fold Change value of the differential expression.

**
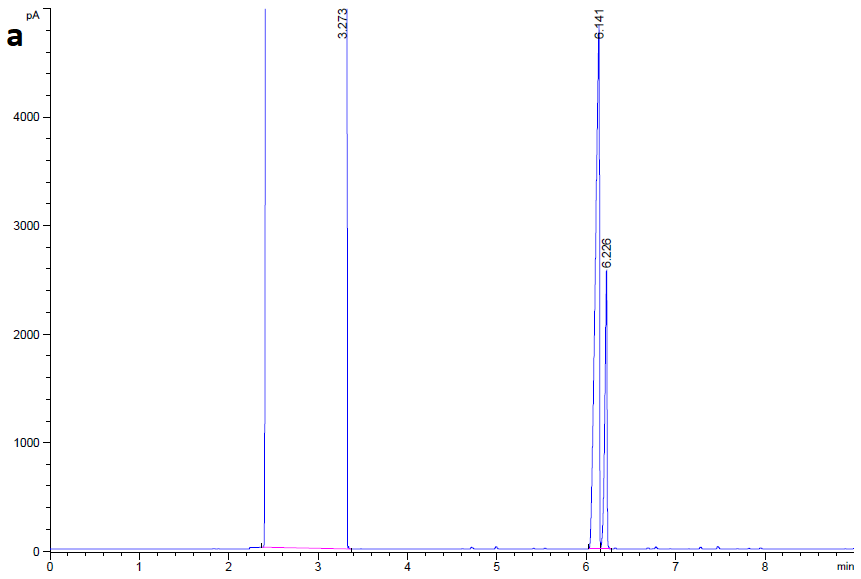
**

**
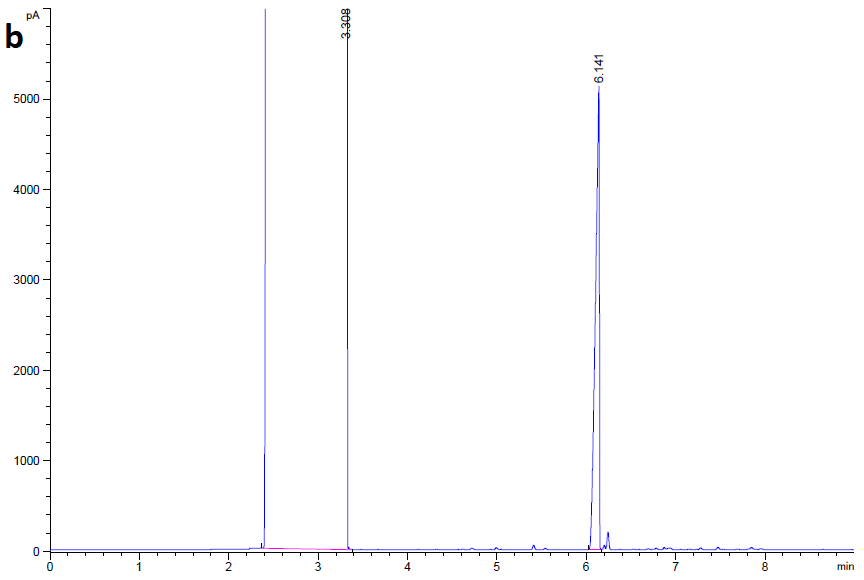
**

**Fig. S3** Quantification of sabinene production by Gas Chromatography. **a** Gas chromatogram of 1 μL sample extracted by cyclohexane from medium containing 100 mg/L commercial sabinene. **b** Gas chromatogram of 1 μL sample extracted by cyclohexane from incubated (XYFHB7) culture without exogenous sabinene.


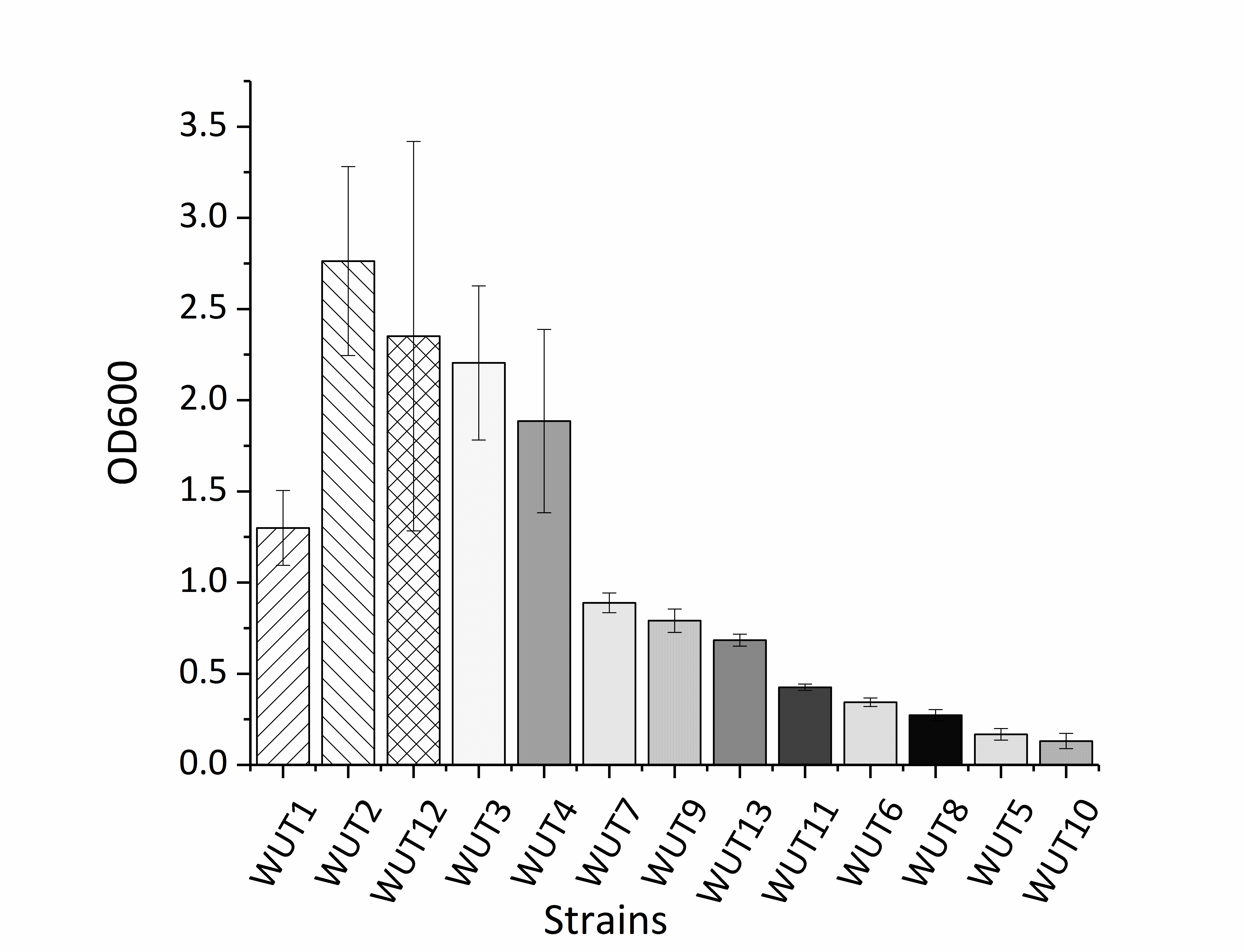


**Fig. S4** Cell density of all the reverse-engineered strains grown after 24 h of cultivation with 0.6 g/L sabinene. Experiments were carried out in triplicate. Error bars represent the standard deviation from the mean.

**Fig. S5** Growth curves of reverse-engineered strains under sabinene stress. Squares with solid lines indicate strain WUT1, expressing empty plasmid pACYCDuet-1. Circles with dashed lines indicate strain WUT2, overexpressing gene *ybcK*. Triangles with dashed lines indicate strain WUT14 (*ΔybcK*), expressing empty plasmid pACYCDuet-1. The experiments were carried out in triplicate. Error bars represent the standard deviation from the mean.

**Fig. S6** Cell length statistics of different strains. BL21(0) represents BL21(DE3) grown without sabinene; BL21(0.6) represents BL21(DE3) grown with 0.6 g/L sabinene; XYF(0.6) represents XYF(DE3) grown with 0.6 g/L sabinene; WUT1(0.6) represents WUT1 grown with 0.6 g/L sabinene; WUT2(0.6) represents WUT2 grown with 0.6 g/L sabinene; WUT3(0.6) represents WUT3 grown with 0.6 g/L sabinene; WUT4(0.6) represents WUT4 grown with 0.6 g/L sabinene. The histogram and error line represent the average length and standard deviation of 50 random cells. The color points in the corresponding position represent the length of each sampled cell.


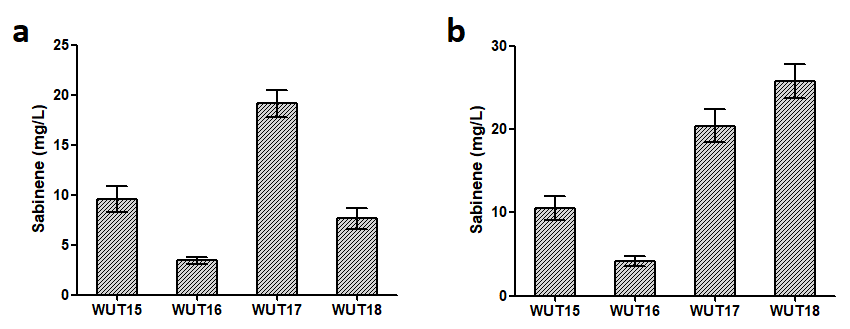


**Fig. S7** Sabinene production of *ybcK*, *scpA* or *ygiZ* overexpression strains. WUT15, BL21(DE3) harboring pHB5, pTrcLower and pET28a-*mvaE-mvaS*, used as a control strain; WUT16, BL21(DE3) harboring pACYC-*ybcK-GPPS2-SabS1*, pTrcLower and pET28a-*mvaE-mvaS*; WUT17, BL21(DE3) harboring pACYC-*scpA-GPPS2-SabS1*, pTrcLower and pET28a-*mvaE-mvaS*; WUT18, BL21(DE3) harboring pACYC-*ygiZ-GPPS2-SabS1*, pTrcLower and pET28a-*mvaE-mvaS*. The experiments were carried out in triplicate. Error bars represent the standard deviation from the mean.

**Table S1** **Other reverse-engineered strains**

| Plasmids/Strains | Description | Reference |
| --- | --- | --- |
| Plasmids |  |  |
| pACYC-*ymgJ* | P15A origin; CmR; P_T7_::*ymgJ* | This work |
| pACYC-*atoD* | P15A origin; CmR; P_T7_::*atoD* | This work |
| pACYC-*kilR* | P15A origin; CmR; P_T7_::*kilR* | This work |
| pACYC-*torY* | P15A origin; CmR; P_T7_::*torY* | This work |
| pACYC-*ybcV* | P15A origin; CmR; P_T7_::*ybcV* | This work |
| pACYC-*ydiM* | P15A origin; CmR; P_T7_::*ydim* | This work |
| pACYC-*bglH* | P15A origin; CmR; P_T7_::*bglH* | This work |
| pACYC-*elaD* | P15A origin; CmR; P_T7_::*elaD* | This work |
| pACYC-*bglF* | P15A origin; CmR; P_T7_::*bglF* | This work |
| Strains |  |  |
| WUT5 | BL21(DE3) harboring pACYC-*ymgJ* | This work |
| WUT6 | BL21(DE3) harboring pACYC-*atoD* | This work |
| WUT7 | BL21(DE3) harboring pACYC-*kilR* | This work |
| WUT8 | BL21(DE3) harboring pACYC-*torY* | This work |
| WUT9 | BL21(DE3) harboring pACYC-*ybcV* | This work |
| WUT10 | BL21(DE3) harboring pACYC-*ydiM* | This work |
| WUT11 | BL21(DE3) harboring pACYC-*bglH* | This work |
| WUT12 | BL21(DE3) harboring pACYC-*elaD* | This work |
| WUT13 | BL21(DE3) harboring pACYC-*bglF* | This work |

**Table S2 Primers used for construction of the reverse-engineered strains and *ΔybcK***

| **Genes** | **Primers** | | **R.enzyme** |
| --- | --- | --- | --- |
| ***ymgJ*** | *ymgJ* -F | 5’- CATGCCATGGGTGAGCAGAAGATATTCATCAGCAACG-3’ | *Nco* I |
|  | *ymgJ* -R | 5’- CCCAAGCTTTCATGATGAAATGATGATATCCCGCAT -3’ | *Hind* III |
| ***atoD*** | *atoD* -F | 5’- CGCGGATCCGATGAAAACAAAATTGATGACATTACAA-3’ | *BamH* I |
|  | *atoD* -R | 5’- CCCAAGCTTTTATTTGCTCTCCTGTGAAACGATGATGT-3’ | *Hind* III |
| ***kilR*** | *kilR*-F | 5’- CGCGGATCCGATGATTGCACATCACTTC-3’ | *BamH* I |
|  | *kilR* –R | 5’- CCCAAGCTTTCACCATGACTCCGC-3’ | *Hind* III |
| ***torY*** | *torY* –F | 5’- CGCGGATCCGATGCGAGGGAAAAAACG-3’ | *BamH* I |
|  | *torY* -R | 5’- CCCAAGCTTCACTGTTTCTCGGTAATATC-3’ | *Hind* III |
| ***ybcV*** | *ybcV* -F | 5’- CGCGGATCCGATGGCTCAGGTTGCC-3’ | *BamH* I |
|  | *ybcV* -R | 5’- CCCAAGCTTTTAGCGTGGAAAGATTTGT-3’ | *Hind* III |
| ***ydiM*** | *ydiM* -F | 5’- CATG CCATGG GCATGAAAAATCCCTATTTCC-3’ | *Nco* I |
|  | *ydiM* -R | 5’- CGCGGATCCTTACCCACCCGGAG-3’ | *BamH* I |
| ***bglH*** | *bglH* -F | 5’- CGCGGATCCGATGTTTAGACGAAATCTTATTACCTCTGCC-3’ | *BamH* I |
|  | *bglH* -R | 5’- CCCAAGCTTTTACCACCAGATTTCAGCCTGGG-3’ | *Hind* III |
| ***elaD*** | *elaD* -F | 5’- CGCGGATCCGATGATGGTTACAGTTGTCAG-3’ | *BamH* I |
|  | *elaD* -R | 5’- CCCAAGCTTTTAACTCACTCTTTTGCCG-3’ | *Hind* III |
| ***bglF*** | *bglF* -F | 5’- CGCGGATCCGATGACGGAGTTAGCCAG-3’ | *BamH* I |
|  | *bglF* -R | 5’- CCCAAGCTTTTAGCGAATGATGGATAACA-3’ | *Hind* III |
| **Donor** | ybcK1 | 5’- ACACGGTTATGGCCATCTG-3’ |  |
| **gRNA** | ybcK2  ybcK3  ybcK4  ybckU1  ybcKD1 | 5’- atccgagatcGGTGACACCTGCTAACGTATG-3’  5’- aggtgtcaccGATCTCGGATTTTAAGTTATGCTATGG-3’  5’- CTAGTGACCTGAAATGCTGCA-3’  5’- cacaccaggtctcaatatgaaggtacACTAGTATTATACCTAGGACTGAGC-3’  5’-cacaccaggtctcaatatcgtgcaagGTTTTAGAGCTAGAAATAGCAAGTTAA-3’ |  |
|  | GA-Insert-F | 5’-ATCATCACCACAGCCAGGAT-3’ |  |
|  | GA-Insert-R | 5’-TTATGCGGCCGCAAGCTT-3’ |  |
|  | GA-Vector-F | 5’-AAGCTTGCGGCCGCATAA-3’ |  |
|  | GA-Vector-R | 5’-ATCCTGGCTGTGGTGATGAT-3’ |  |
|  | GA-mvaES-F | 5’-AAGGAGATATACCATGGAGGAGG-3’ |  |
|  | GA-mvaES-R | 5’-CTGCAGTTAGTTTCGATAAGAGC-3’ |  |
|  | GA-28a-F | 5’-tatcgaaactaactgcagCTCGAGCACCACCACCA-3’ |  |
|  | GA-28a-R | 5’-CCTCCTCCATGGTATATCTCCTTCTTAAAG-3’ |  |

**Table S3 The top 100 up-regulated genes**

| GeneSymbol | length | log2 FoldChange | p-value adjusted | Description |
| --- | --- | --- | --- | --- |
| *yjjZ* | 237 | 6.077672383 | 3.18E-20 | Uncharacterized protein |
| *yrhA* | 114 | 5.698185099 | 1.76E-11 | Uncharacterized protein |
| *rrlD* | 2904 | 5.43105452 | 4.45E-09 | 23S ribosomal RNA |
| *ydeS* | 531 | 5.293943103 | 8.51E-08 | Predicted fimbrial-like adhesin protein |
| *ariR* | 267 | 5.169961415 | 8.50E-07 | Probable RcsB/C two-component-system connector |
| *ymgJ* | 186 | 5.157986885 | 1.94E-09 | General stress-inducible proteinsc |
| *rrlG* | 2904 | 5.028056637 | 7.06E-08 | 23S ribosomal RNA |
| *yraI* | 696 | 5.010418677 | 4.06E-09 | Putative fimbrial chaperone |
| *ygiZ* | 333 | 4.775909937 | 3.54E-12 | Inner membrane protein |
| *rrlA* | 2905 | 4.764354782 | 1.00E-07 | 23S ribosomal RNA |
| *rrsD* | 1542 | 4.686717582 | 7.99E-11 | 16S ribosomal RNA |
| *yccE* | 1257 | 4.392377943 | 2.02E-07 | Uncharacterized protein |
| *yceO* | 141 | 4.314737308 | 1.01E-08 | Uncharacterized protein |
| *atoD* | 663 | 4.231411852 | 1.89E-09 | Acetate CoA-transferase subunit alpha |
| *entC* | 1176 | 4.227434989 | 3.78E-12 | Isochorismate synthase |
| *yibV* | 111 | 4.190591723 | 1.58E-10 | Uncharacterized protein |
| *psuK* | 942 | 4.129336642 | 1.45E-08 | Putative pseudouridine kinase |
| *yncH* | 213 | 4.097164763 | 9.04E-09 | Uncharacterized protein |
| *rhaA* | 1260 | 4.076578063 | 2.02E-08 | L-rhamnose isomerase |
| *prpB* | 891 | 4.041804668 | 4.06E-09 | 2-methylisocitrate lyase |
| *ycgX* | 405 | 3.948370444 | 4.32E-09 | Uncharacterized protein |
| *yncI* | 315 | 3.945334564 | 4.06E-09 | Putative transposase |
| *ybcK* | 1527 | 3.939606713 | 7.99E-11 | DLP12 prophage |
| *entE* | 1611 | 3.932331551 | 6.58E-11 | Enterobactin synthase component E |
| *kilR* | 222 | 3.931475332 | 1.00E-07 | Killing protein; Rac prophage; inhibitor of FtsZ |
| *torY* | 1101 | 3.907124887 | 3.29E-08 | Cytochrome c-type protein |
| *csgE* | 390 | 3.862800387 | 9.51E-07 | Curli production assembly/transport component |
| *arpB* | 1476 | 3.861588932 | 2.39E-10 | Putative ankyrin repeat protein B |
| *yhiD* | 648 | 3.854987708 | 2.02E-08 | Putative magnesium transporter |
| *ompL* | 693 | 3.848059972 | 1.40E-06 | Porin |
| *yjbS* | 204 | 3.807798404 | 5.51E-07 | Uncharacterized protein |
| *ybeR* | 708 | 3.803613905 | 1.69E-06 | Uncharacterized protein |
| *yeeL* | 1053 | 3.799846224 | 4.32E-09 | Uncharacterized protein |
| *yqiI* | 1065 | 3.790838159 | 3.07E-10 | Putative fimbrial protein |
| *yaiV* | 624 | 3.779354343 | 8.54E-07 | Predicted DNA-binding transcriptional regulator |
| *rclC* | 594 | 3.720285496 | 7.86E-10 | Inner membrane protein |
| *fimI* | 540 | 3.715962798 | 4.11E-08 | Putative fimbrial protein |
| *yddK* | 381 | 3.713847282 | 1.01E-07 | Leucine-rich repeat domain-containing protein |
| *yhhZ* | 1179 | 3.699891136 | 2.81E-11 | Uncharacterized protein |
| *yjcF* | 1293 | 3.694472672 | 4.64E-08 | Uncharacterized protein |
| *yahL* | 816 | 3.614688873 | 7.69E-08 | Uncharacterized protein |
| *rhsH* | 708 | 3.61162449 | 1.79E-08 | Uncharacterized protein |
| *ynbB* | 897 | 3.609279522 | 1.31E-06 | Putative CDP-diglyceride synthase |
| *ydeT* | 1227 | 3.594073281 | 8.74E-07 | Fimbrial usher domain-containing protein |
| *ttdR* | 933 | 3.58145119 | 1.21E-09 | HTH-type transcriptional activator |
| *ydcC* | 1137 | 3.555275596 | 1.52E-06 | H repeat-associated putative transposase |
| *ibsE* | 60 | 3.547726781 | 9.51E-07 | Small toxic protein |
| *acrS* | 663 | 3.542067864 | 5.64E-07 | acrAB operon transcriptional repressor |
| *ecpR* | 591 | 3.519948149 | 4.45E-09 | HTH-type transcriptional regulator |
| *yrhC* | 351 | 3.515486326 | 3.67E-07 | Uncharacterized protein |
| *yhaB* | 540 | 3.512051072 | 2.46E-08 | Uncharacterized protein |
| *ybcV* | 411 | 3.510490058 | 6.46E-08 | DLP12 prophage |
| *yqiH* | 750 | 3.503092768 | 1.28E-07 | Putative fimbrial chaperone |
| *yqcE* | 1278 | 3.476869813 | 8.54E-07 | Inner membrane protein |
| *wcaE* | 747 | 3.466168229 | 2.35E-06 | Putative colanic acid biosynthesis glycosyltransferase |
| *nanC* | 717 | 3.462750254 | 2.85E-07 | N-acetylneuraminic acid outer membrane channel protein |
| *bdm* | 216 | 3.42504654 | 1.50E-07 | Biofilm-dependent modulation protein |
| *ygcW* | 786 | 3.415339657 | 5.80E-07 | Putative deoxygluconate dehydrogenase |
| *sfmH* | 984 | 3.410275657 | 1.42E-06 | Putative fimbrial adhesin protein |
| *ybbW* | 1455 | 3.391439293 | 1.12E-06 | Putative allantoin permease |
| *elfD* | 702 | 3.388374261 | 1.02E-07 | Putative fimbrial chaperone |
| *yhiS* | 483 | 3.364852651 | 9.04E-09 | Uncharacterized protein |
| *bglG* | 837 | 3.362053132 | 2.76E-07 | Cryptic beta-glucoside bgl operon antiterminator |
| *xylF* | 993 | 3.345550429 | 1.52E-08 | D-xylose transport system substrate-binding protein |
| *yiiE* | 213 | 3.323634971 | 1.14E-06 | Putative DNA-binding transcriptional regulator |
| *ydiN* | 1266 | 3.29137742 | 1.75E-06 | Inner membrane transporter |
| *fepE* | 1134 | 3.278440685 | 1.01E-08 | Ferric enterobactin transport protein |
| *arpA* | 2187 | 3.275657549 | 3.41E-08 | Regulator of acetyl CoA synthetase |
| *ydiM* | 1215 | 3.217260596 | 4.96E-07 | Inner membrane transporter |
| *ycjM* | 1680 | 3.210328912 | 2.04E-06 | Glucosylglycerate phosphorylase |
| *ygjJ* | 1071 | 3.210035245 | 2.17E-07 | Uncharacterized protein |
| *csgD* | 651 | 3.176333655 | 1.87E-06 | csgAB operon transcriptional regulatory protein |
| *bglH* | 1617 | 3.151768069 | 8.75E-09 | Cryptic outer membrane porin |
| *yjjQ* | 726 | 3.105866235 | 8.68E-07 | Putative transcription factor |
| *yihR* | 927 | 3.097458398 | 1.40E-06 | Putative aldose 1-epimerase |
| *fes* | 1203 | 3.075814508 | 9.04E-09 | Enterochelin esterase |
| *yjhZ* | 246 | 3.072639086 | 2.37E-06 | Uncharacterized protein |
| *elaD* | 1212 | 3.070684375 | 4.65E-08 | Protease |
| *ycgH* | 75 | 3.069417775 | 1.35E-06 | Uncharacterized protein |
| *yggR* | 981 | 3.059501027 | 8.90E-07 | Type II/IV secretion system family protein |
| *bglF* | 1878 | 3.039815419 | 1.48E-07 | PTS system beta-glucoside-specific EIIBCA component |
| *motB* | 927 | 3.031306433 | 1.62E-06 | Motility protein |
| *ycjT* | 2268 | 3.02141439 | 5.14E-07 | Kojibiose phosphorylase |
| *yhhI* | 1137 | 3.010251762 | 3.73E-07 | Putative H repeat-associated transposase |
| *emrY* | 1539 | 3.004854958 | 1.67E-06 | Multidrug resistance protein |
| *agaI* | 756 | 3.000251823 | 2.28E-07 | Putative deaminase |
| *yihQ* | 2037 | 2.99862945 | 1.50E-08 | Sulfoquinovosidase |
| *dgoD* | 1149 | 2.99595596 | 8.62E-08 | D-galactonate dehydratase |
| *recE* | 2601 | 2.963990152 | 1.12E-06 | Exodeoxyribonuclease |
| *yliE* | 2349 | 2.865139051 | 1.12E-06 | Cyclic di-GMP phosphodiesterase |
| *uacT* | 1449 | 2.853936782 | 2.29E-06 | Uric acid transporter |
| *alsA* | 1533 | 2.852248011 | 8.71E-07 | D-allose import ATP-binding protein |
| *ygcG* | 873 | 2.842955225 | 1.28E-07 | Uncharacterized protein |
| *ecpA* | 588 | 2.780058934 | 1.72E-06 | Common pilus major fimbrillin subunit |
| *yiiG* | 1056 | 2.772336276 | 9.51E-07 | Uncharacterized protein |
| *mdtQ* | 1287 | 2.768792294 | 1.63E-07 | Multidrug resistance outer membrane protein |
| *sfmD* | 2604 | 2.758867149 | 2.22E-06 | Outer membrane usher protein |
| *gspC* | 816 | 2.727291702 | 1.65E-06 | Type II secretion system protein |
| *caiT* | 1515 | 2.706170099 | 1.92E-06 | L-carnitine/gamma-butyrobetaine antiporter |
| *yjhR* | 1029 | 2.59018347 | 2.18E-06 | Putative frameshift suppressor |
